# Efficacy and safety of PDE5 inhibitors in the treatment of diabetes mellitus erectile dysfunction: Protocol for a systematic review

**DOI:** 10.1097/MD.0000000000012559

**Published:** 2018-10-05

**Authors:** Xiao Li, Qi Zhao, Jingshang Wang, Jisheng Wang, Hengheng Dai, Haisong Li, Bin Wang

**Affiliations:** a Department of Andrology, Dongzhimen Hospital, Beijing University of Chinese Medicine, Dongcheng District; b Department of Traditional Chinese Medicine, Beijing Obstetrics and Gynecology Hospital, Capital Medical University, Chaoyang District, Beijing, China.

**Keywords:** diabetes mellitus erectile dysfunction, PDE5 inhibitors, protocol, systematic review

## Abstract

**Introduction:**

Diabetes mellitus erectile dysfunction (DMED) is a common complication of long-term hyperglycemia. With the increasing of diabetic patients, the number of DMED patients is gradually growing up, which has a serious impact on the quality of life of patients. PDE5 inhibitors have good clinical efficacy in DMED patients. This study is designed to evaluate the efficacy and safety of PDE5 inhibitors in DMED patients.

**Methods and analysis:**

We will systematically search all randomized controlled trials (RCTs) by electronic and manual search. Electronic retrieval of the database includes Pubmed, EMBASE, The Cochrane Library, the Chinese BioMedical Literature Database, the China National Knowledge Infrastructure (CNKI), the China Science and Technology Journal database (VIP) and the Wanfang database. Manual search will retrieve gray literature, including dissertations, ongoing experiments, grey literature, conference and unpublished documents. We use the IIEF-5 scale as the primary outcome of DMED. We also need to pay attention to the following outcomes: the sexual satisfaction of patients and their partners, like IIEF Q3 Q4; SEP 2, 3; GAQ. More importantly, the adverse reactions of patients during medication will also be taken seriously. Two reviewers will independently read the articles, extract the data information, and give the assessment of risk of bias. Data analysis will be used the special software like RevMan (version 5.3.5), ENDNOTE X7 and STATA 13.

**Results:**

This study will provide a comprehensive assessment based on current evidence of PDE5 inhibitors for DMED, especially its impacts on International Index of Erectile Function, the sexual satisfaction of patients and their partners and safety.

**Ethics and dissemination:**

This systematic review will evaluate the efficacy and safety of PDE5 inhibitors on DMED. This review does not require ethical approval and will be reported in a peer-reviewed journal.

**Trial registration number:**

PROSPERO CRD42018095185.

## 1 Introduction

Diabetes mellitus erectile dysfunction (DMED) is one of the common chronic complications of diabetes mellitus (DM), especially in patients with type 2 diabetes.^[1–3]^ Erectile dysfunction (ED) is a common sexual dysfunction in men.^[4]^ It means that when attempting sexual intercourse, the hardness of penile erection is insufficient to insert into the vagina, or the penile cannot maintain or maintain a full erection to obtain a satisfactory sex life.^[5]^ It is affected by various factors such as vascular, neurological, psychological, and endocrine hormones.^[6–9]^ According to the report, the incidence of ED in patients with DM is 1.9 to 5 times that of non-DM patients and 35% to 90% of people with diabetes worldwide are affected by ED.^[5,10]^ As the largest diabetes country in China, China is expected to reach 40 million DMED patients by 2025. DMED brings serious psychological and economic burden to patients and has a great impact on the quality of life of patients. ^[11]^

Pharmacotherapy is the primary treatment for ED, including PDE5 inhibitors, androgen therapy, and vasoactive agents.^[12–15]^ Phosphodiesterase-5 (PDE5) inhibitors, the first-line oral drugs recommended by World Health Organization (WHO) for the treatment of ED, have also begun to be widely used in the treatment of DMED, included Sildenafil, Tadalafil, Vardenafil, and so on.^[16–18]^ The medicine can mainly inhibit PDE5, expressed in the corpus cavernous, to increase cGMP concentration in vascular smooth muscle cells, decrease intracellular calcium concentration, cause smooth muscle relaxation and increase cavernous blood flow which could improve erectile situation.^[19]^ Studies have shown that PDE5 inhibitors treatment of DMED can improve the International Index of Erectile Function-5 (IIEF-5) and sexual success rate in a considerable number of patients.^[20,21]^ Although meta-analyses have shown that PDE5 inhibitors can safely and effectively treat ED, whether they are still safe and effective for DMED with more complex etiologies remains to be assessed.^[22,23]^ Therefore, this review hopes evaluate the efficacy and safety of PDE5 inhibitors in the treatment of DMED to provide the newest evidence for clinical.

## 2 Methods

The protocol has been registered on PROSPERO as CRD42018095185. (https://www.crd.york.ac.uk/prospero/display_record.php?RecordID=95185) The protocol has obeyed from Preferred Reporting Items for Systematic Reviews and Meta-Analyses Protocols (PRISMA-P) statement guidelines. We will document the essential protocol amendments in the full review.

### 2.1 Inclusion criteria for study selection

#### 2.1.1 Types of studies

Randomized controlled trials (RCTs) of PDE5 inhibitors for the treatment of DMED will be included, with language limited to Chinese and English. Observational studies, non-randomized controlled studies, case report will be excluded.

#### 2.1.2 Types of participants

The cases selected are adult male DMED patients over 18 years old. Besides, the region, nation, ethnic and sources of cases are not limited.

#### 2.1.3 Types of interventions

##### 2.1.3.1 Experimental interventions

The treatment group will use the PDE5 inhibitors, including avanafil, lodenafil, mirodenafil, sildenafil, tadalafil, udenafil, or vardenafil, with no limited of the dose and frequency of the medicine. The trial period requires more than 1 course of treatment.

##### 2.1.3.2 Control interventions

The intervention group was placebo.

The control group will be placebo. The following treatment comparisons will be investigated:

1. sildenafil versus placebo
2. avanafil versus placebo
3. lodenafil versus placebo
4. mirodenafil versus placebo
5. tadalafil versus placebo
6. udenafil versus placebo
7. vardenafil versus placebo.

#### 2.1.4 Types of outcome measures

##### 2.1.4.1 Primary outcomes

We use the IIEF-5 scale as the primary outcome of DMED.

##### 2.1.4.2 Secondary outcomes

We also care about the following indexes: the sexual satisfaction of patients and their partners, like IIEF Q3 Q4; SEP 2, 3; GAQ. More importantly, the adverse reactions of patients during medication will be taken seriously.

### 2.2 Search methods for the identification of studies.

#### 2.2.1 Electronic searches

The literature research will be divided into electronic search and manual search, by the time of August 31, 2018. Electronic search databases include Pubmed, EMBASE, The Cochrane Library, the Chinese BioMedical Literature Database, the China National Knowledge Infrastructure (CNKI), the China Science and Technology Journal database (VIP), and the Wanfang database. For a more complete search of the database, the team members will develop an elaborate search strategy based on the Cochrane Handbook Guidelines. The search terms used in the systematic review will include PDE5 inhibitors, avanafil, lodenafil, mirodenafil, sildenafil, tadalafil, udenafil, vardenafil, diabetes mellitus, diabetes and ED. Chinese translations of these search terms will be used to search in Chinese databases. Search strategy for Medline is shown in Table 1.

**Table 1:**
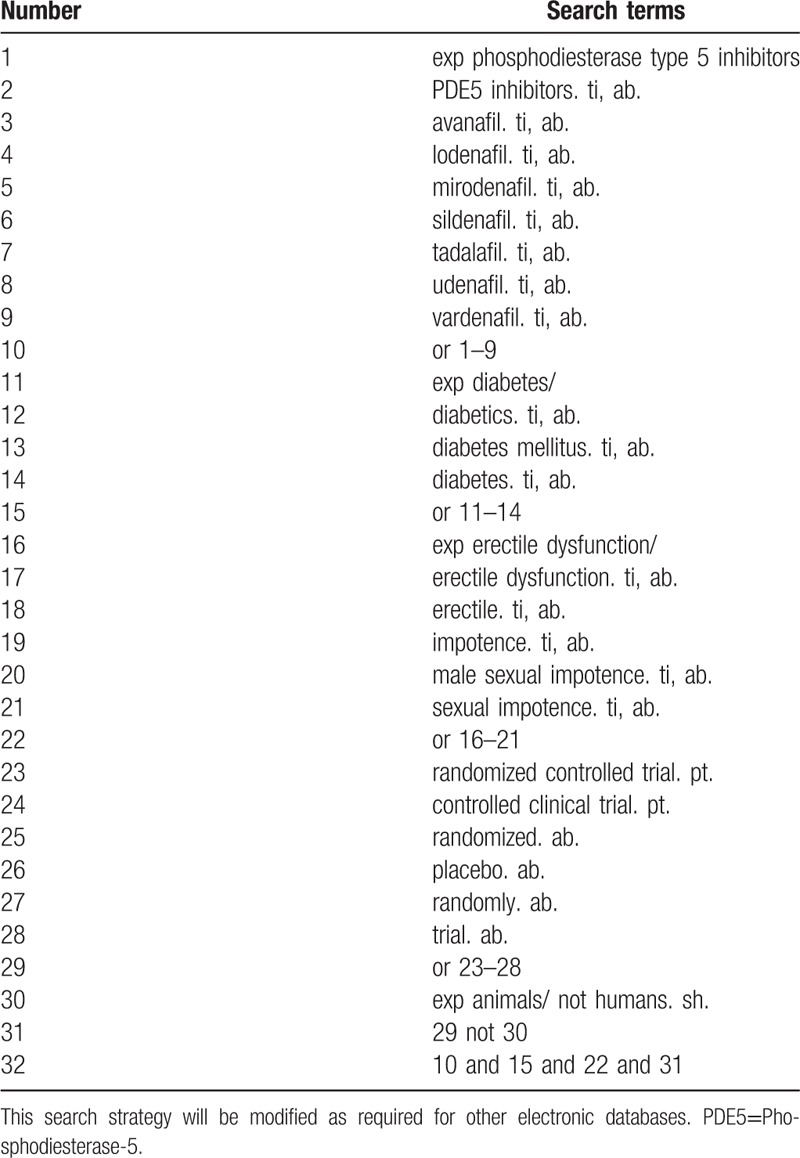
Search strategy used in PubMed database.

#### 2.2.2 Searching other resources

Manual searches mainly retrieve dissertations, ongoing experiments, grey literature, conference and unpublished documents. First, we will search for the abstracts of them associated with ED and diabetes milltus, and then try to acquire the full passage if necessary. Ongoing trials which relevant to this term will be retrieved from the clinical registration platform, like the WHO International Clinical Trials Registry Platform (ICTRP), the Chinese Clinical Trial Registry and ClinicalTrials.gov. We will manually search OpenGrey.eu for gray literature and ongoing trials in clinical trials.

### 2.3 Data collection and analysis

#### 2.3.1 Selection of studies

**Figure 1:**
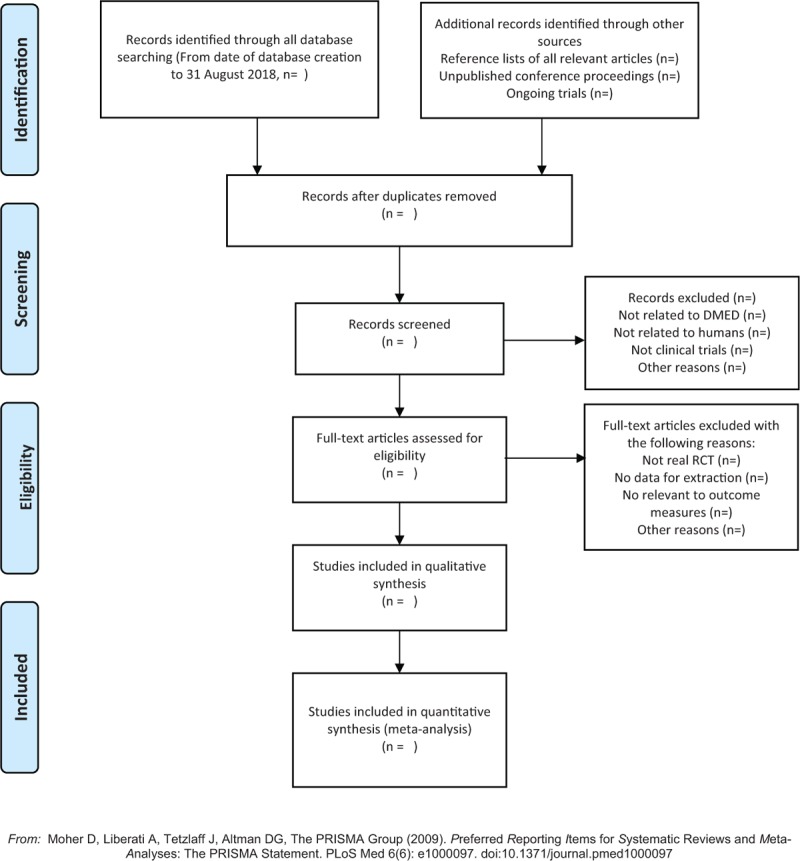
The PRISMA flow chart. PRISMA=Preferred Reporting Items for Systematic Reviews and Meta-Analyses.

Before the search begins, each reviewer will receive professional training to ensure consistency in the selection process and avoid the risk of bias (ROB) in human factors. The screening process will use EndNote X7 literature management software. The 2 review authors (XL and QZ) will read the topics, abstracts, keywords, and the full text independently if needed. The screening process will be strictly in accordance with the inclusion criteria. If there is a disagreement, it will be decided with another reviewer (HL). We will record the exclusion reason for the excluded literature. The details of selection process will be shown in the Preferred Reporting Items for Systematic Reviews and Meta-Analyses (PRISMA) flow chart (Figure 1)

#### 2.3.2 Data extraction and management

Two reviewers (JW and JS) will record the relevant information needed from the documents that have already been included. This information will form a detail extraction form. If the details in the literature are incomplete, we will contact the author via email, telephone, and so on. The following data will be extracted:

1. General information: research identification, publication time, title of article, correspondent author, contact information.
2. Study methods: study design, sample size, randomization method, allocation concealment, blinding, incomplete report or selecting report, other sources of bias.
3. Participants: inclusion criteria and exclusion criteria, age of patients, gender, diabetes mellitus diagnostic criteria, ED diagnostic criteria, severity, race, research site, baseline of erectile function.
4. Interventions: types of PDE5 inhibitors, the dose of the medicine, treatment duration, and frequency.
5. Outcomes: IIEF-5, the sexual satisfaction of patients and their partners, like IIEF Q3 Q4; SEP 2, 3; GAQ and safety outcomes.
6. Notes: financial support, conflicts of interest, ethical approval, important citations.

#### 2.3.3 Assessment of ROB in included studies

The quality assessment of the literature will be assessed using the ROB assessment tool in the Cochrane. Evaluation criteria include random sequence generation, allocation concealment, blinding of outcome assessment, incomplete outcome data, selective reporting and others bias. Two reviewers (JW and HD) will evaluate each article independently and give a low bias, unclear, or high bias evaluation. If there is a disagreement among reviewers, it will be discussed with another reviewer (BW) to make a decision.

#### 2.3.4 Measures of treatment effect

Mean difference (MD) or standardized mean difference (SMD) and 95% confidence interval (CI) will be recorded for continuous variable outcome. For dichotomous outcomes, we will record the relative risk (RR) and 95% CI.

#### 2.3.5 Unit of analysis issue

If the literature included contains cross-over trials, we will only evaluate the first experimental period data to avoid carry-over effects. For multiple intervention groups, we will combine respectively all the experimental groups and control groups to prevent a unit of analysis issue.

#### 2.3.6 Dealing with missing data

If the data in the included literature is missing, we will first analyze the cause of the data loss. After that, we will contact the corresponding author of the article in as many ways as possible, by telephone, email, and so on. If the data is still not available, we will analyze the reason in the discussion.

#### 2.3.7 Assessment of heterogeneity

Only a complete trial results will be used for data analysis. Heterogeneity is represented by *I*^2^. When the data results meet the meta-analysis requirements, the results will be merged. When *I*^2^ ≤ 50% and *P* ≥.1, a fixed-effect model will be used and a random-effects model will be used when *I*^2^ > 50% or *P* < .1.

#### 2.3.8 Data synthesis and analysis

We will use Review Manager Software (RevMan V.5.3.5) provided by Cochrane Collaboration for data synthesis and analysis. When *I*^2^ < 50%, a fixed-effects model will be used to calculate the RR and MD. When *I*^2^ ≥50%, we will use a random-effects model to synthesize the data. If the heterogeneity is high, we will analyze the cause of the heterogeneity and subgroup analysis will be performed. If meta-analysis is not appropriate, we may use narrative synthesis and describe it in the discussion.

#### 2.3.9 Assessment of publication bias

When the primary outcome measure will be able to include a sufficient number of documents (≥10 articles), the “funnel plot” was used to detect the risk of publication bias. When the number is less than 10, the publication bias assessment will be determined by Begg test and Egger test using Stata 13.0 (StataCorp., College Station, Texas) software.

#### 2.3.10 Subgroup analysis

When there is significant heterogeneity in the meta-analysis, subgroup analysis is performed on different interventions, controls and outcome measures.

#### 2.3.11 Sensitivity analysis

We will try to perform sensitivity analysis for primary outcomes to examine the robustness of conclusions, and we will still evaluate the impact of methodological quality, sample size and missing data.

#### 2.3.12 Grading the quality of evidence

When the meta-analysis is completed, the Grading of Recommendations Assessment (GRADE) pro online software will be used to evaluate the quality of included studies. The evaluation included: bias risk; heterogeneity; indirectness; imprecision; publication bias. And each evidence will be decided for “very low”, “low”, “moderate”, or “high” judgment.

## 3 Discussion

Diabetes has a variety of chronic complications, including heart disease, hypertension, stroke, ED, and so on.^[24]^ Studies show that the occurrence of diabetes is most closely associated with ED.^[25,26]^ Generally, the penis erection is the release of neurotransmitters from cavernous nerve endings under sexual stimulation, acting on the smooth muscles of the penis to dilate and congest the penis erection. As we all know, the various chronic complications of the diabetes are mainly due to damage to the function of autonomic nerves and blood vessels. At present, most scholars believe that the release of nitric oxide (NO) from endothelial cells is the main neurotransmitter that causes penis erection. The important receptor for NO is sGC, therefore, NO-sGC-cGMP mediates endothelium-dependent vascular expansion effect pathway.^[27–29]^ All affect the above vasodilator effect any link in the pathway can lead to ED. PDE5 is inactivated by competitive inhibition of cGMP and PDE5 binding, reduce cGMP hydrolysis, thereby increasing cGMP concentration, and finally close fine the calcium channel on the cell membrane. When intracellular calcium concentration drops, smooth muscle cells dilate and the penis is congested, so the erectile effect increases.^[30]^

At present, there are many reports that the PDE5 inhibitors have a significant curative effect for DMED and the incidence of adverse events is low. We hope that this systematic review will provide more clinical evidence to facilitate the use of clinicians. However, this review still has some limitations and needs to be continuously improved in the future. First, there may be a certain ROB as language limited by Chinese and English. Second, different duration and frequency of medicine, the age of patients and degree of DMED may cause high heterogeneity.

## Author contributions

XL, QZ, JSW, JSW and HHD contributed equally to this work and are co-first. XL and JW designed the systematic review. QZ and JW drafted the protocol and JW and HD revised the manuscript. XL and JW will independently screen the potential studies, extract data and finish data synthesis. BW, JW will assess the ROB. BW and HL will arbitrate any disagreement and ensure that no errors occur during the review.

**Conceptualization:** Haisong Li, Bin Wang.

**Data curation:** Jingshang Wang, Jisheng Wang.

**Investigation:** Xiao Li, Qi Zhao.

**Software:** Hengheng Dai.
