# Efficacy and safety of PDE5 inhibitors in the treatment of diabetes mellitus erectile dysfunction: Protocol for a systematic review: Erratum

**DOI:** 10.1097/MD.0000000000013203

**Published:** 2018-11-02

In the article, “Efficacy and safety of PDE5 inhibitors in the treatment of diabetes mellitus erectile dysfunction: Protocol for a systematic review”,^[1]^ which appeared in Volume 97, Issue 40 of *Medicine*, the footnote should read: XL, QZ, JSW, JSW and HHD contributed equally to this work and are co-first authors.

Dr. Jingshang Wang should be affiliated with both affiliations a and b.

The corresponding author information should appear as: Bin Wang, Haisong Li, Department of Andrology, Dongzhimen Hospital, Dongcheng District, Hai Yun Cang on the 5th Zip, Beijing 100029, China (e-mails: dayiwangbin@sina.com, 1028bj@sina.com).
